# Gamma-Irradiation-Induced Degradation of the Water-Soluble Polysaccharide from *Auricularia polytricha* and Its Anti-Hypercholesterolemic Activity

**DOI:** 10.3390/molecules27031110

**Published:** 2022-02-07

**Authors:** Ping Li, Chuan Xiong, Wenli Huang

**Affiliations:** 1 Biotechnology and Nuclear Technology Research Institute, Sichuan Academy of Agricultural Sciences, Chengdu 610066, China; liping_0901@sina.com (P.L.); xiongchuan1234@126.com (C.X.); 2 Key Laboratory of Coarse Cereal Processing, Ministry of Agriculture and Rural Affairs, School of Food and Biological Engineering, Chengdu University, Chengdu 610106, China

**Keywords:** *Auricularia polytricha*, polysaccharide, gamma irradiation, degradation, anti-hypercholesterolemic

## Abstract

The water-soluble polysaccharides (APPs) isolated from the edible mushroom *Auricularia polytricha* were irradiated by γ-ray at doses of 10, 100, and 1000 kGy. The effect of gamma irradiation on the degradation of the polysaccharide was investigated. After irradiation treatment, the viscosity and molecular weight of APPs decreased with the increase in the irradiation dose. The changes in the enthalpy of APPs after irradiation treatment were observed. Meanwhile, SEM showed that R-APPs were crushed into fragments and the surfaces became smooth and wrinkled after irradiation. In further spectrum analysis, it was found that the glycoside bonds of the polysaccharides were broken and accompanied by the formation of double bonds. This suggested that gamma irradiation could cause the depolymerization and oxidation of polysaccharides. In addition, irradiated APPs could reduce the body weight of hyperlipidemia mice. The levels of serum and liver TC, TG, and serum LDH-c significantly decreased in hyperlipidemia mice after treatment by irradiated APPs. It indicated that gamma irradiation significantly improved the anti-hypolipidemic activity of APPs. The relationship between the physicochemical properties and hypolipidemic activity of polysaccharides was interpreted, which provides a theoretical basis for the further development of APP products. Gamma irradiation is a viable technology for macromolecular modification for degradation.

## 1. Introduction

*Auricularia polytricha*, a traditional and popular edible mushroom in China, is well known for its low fat and high dietary fiber contents. *A. polytricha* is not only rich in nutrients; it also has many medicinal ingredients. It could be used as a candidate of complementary and alternate medicine (CAM) in China [1]. Polysaccharide is one of the most important constituents in mushroom, and is responsible for various pharmacological properties. The salt-soluble polysaccharide SSP from the mycelia of *A. polytricha* exhibited antimutagenic activity against the DNA-damaging effect in vivo [2]. A fraction of polysaccharides APPIIA showed significant antitumor effects and regulated macrophage activation [3]. The water-soluble polysaccharide components from fruit bodies of *A. polytricha* exhibited significant anticancer and antioxidant activities [4]. The polysaccharides from many plants and mushrooms were found to be effective in lowering the lipid levels in hypercholesterolemic or hyperlipidemic mammals. A polysaccharide fraction isolated from *Enteromorpha prolifera* had a high hypolipidemic antioxidant activity [5]. Huang et al. reported that the polysaccharide from fuzi showed a cholesterol-lowering effect in hypercholesteremic rats [6]. The oral administration of polysaccharides from *Auricularia auricular* significantly lowered the serum total cholesterol (TC) and low-density lipoprotein cholesterol (LDH-c) and enhanced the lipoprotein lipase activity in ICR mice [7]. Recently, it was found that a soluble polysaccharide isolated from *A. polytricha* could recover the serum lipid level in cholesterol-enriched diet-induced hypercholesterolemic mice back to normal [8].

The biological activities of polysaccharides are closely related to the complex structure and physical characteristics. The molecular weight (Mw) of polysaccharide from mushrooms and plants is one of the crucial factors affecting its biological activity. Four polysaccharides with different molecular weights and monosaccharide compositions from *Astragalus* exhibited various immunobiological activities [9]. Generally, the polysaccharides with low molecular weight showed stronger biological activities. Polysaccharide with high molecular weight was always accompanied with high viscosity and low solubility, which restricted diffusion into biological tissue. Low-molecular-weight laminarin inhibited tumor cell proliferation, but the original high-molecular-weight laminarin had little effect [10]. It was previously reported that the bioactivity of polysaccharide from *Grifola frondose* [11], seaweeds [12], and oat [13] was enhanced after partial degradation.

Many methods, such as acid–base hydrolysis, enzymolysis, radiolysis, and ultrasonic and microwave degradation have been used to produce low-molecular-weight polysaccharide. Gamma irradiation is a simple and viable technology for reducing the average molecular weight of polysaccharide. It could lead to the degradation of polysaccharide and changes in biological activities, resulting in decreases in molecular weight, viscosity, and rheological properties [14]. The average molecular weight and viscosity of the beta-glucan from *Agaricus bisporus* decreased with the increases in irradiation doses [15]. Gamma irradiation induced the degradation of laminarin with structural changes, decreasing the average molecular weight, and exhibiting a stronger antioxidant activity [16]. Melanin synthesis inhibitory activities of laminarin were enhanced by gamma irradiation [17]. Moreover, gamma irradiation caused changes in bean polysaccharides in the physicochemical morphological properties and increases in antioxidant activities [18].

The polysaccharide of *A. polytricha* had properties of a high molecular weight, high viscosity, and an insoluble fraction portion. In this study, the effect of gamma irradiation on the physicochemical properties and the antihyperlipidemic activity of the polysaccharide from *A. polytricha* were investigated.

## 2. Results and Discussion

### 2.1. Effect of Gamma Irradiation on the Physicochemical Properties of APPs

**Figure 1:**
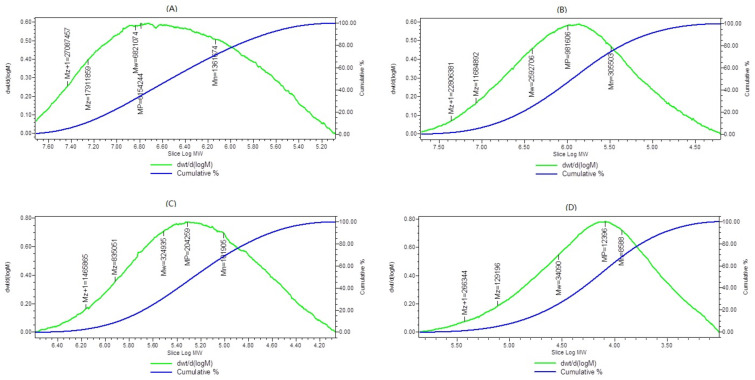
The molar mass distribution of APP and irradiated APP. (**A**) Native APP; (**B**) 10 kGy; (**C**) 100 kGy; (**D**) 1000 kGy.

Gamma irradiation could cause the degradation of polysaccharides from *A. polytricha* (APPs). The molar mass distribution of APP and irradiated APP was calculated through gel permeation chromatography (Figure 1). The effects of irradiated dose on the average Mw of polysaccharide solution are shown in Table 1. The average molecular weight of the native APPs was 6820 kD with high viscosity. The average molecular weight gradually decreased to 2590 kD, 325 kD, and 34 kD after irradiation at doses of 10 kGy (R-APP1), 100 kGy (R-APP2), and 1000 kGy (R-APP3), respectively. It was found that polysaccharides with high molecular weight were always accompanied by high viscosity and poor solubility [19]. Herein, the viscosity of native APPs solution was 132.65 cP. After irradiation treatment, the viscosity of the polysaccharide solution decreased with the decrease in molecular weight. The viscosity of APPs irradiated at a dose of 1000 kGy (R-APP3) dropped by nearly 90% compared with the native APPs. The reduction in viscosity and Mw confirmed that gamma irradiation could cause the degradation of APPs. This was consistent with previous reports that the average molecular weights of beta-glucan [13], kappa-carrageenan [20], glucomannan [21], laminarin [17] decreased after gamma irradiation. The apparent viscosity of exopolysaccharide solution from *Xanthomonas campestris* decreased with the increasing irradiation dose [22]. Additionally, the reduction in polydispersity of APPs was observed after gamma irradiation, which indicated that APPs with high-weight molecules degraded into uniform ones [23]. Notably, the polydispersity of APPs treated by irradiation at 10 kGy (R-APP1) was much higher than that of native APPs. It suggested that the partial degradation or crosslink might occur when APPs were irradiated at relatively low doses, which was responsible for the non-uniformity [24]. Gamma irradiation of aqueous κ-carrageenan solution produces a heterogeneous mixture of low-MW degradation products [20]. The polydispersity of the polysaccharide would decrease because of further full degradation by gamma radiation with a higher dose.

**Figure 2:**
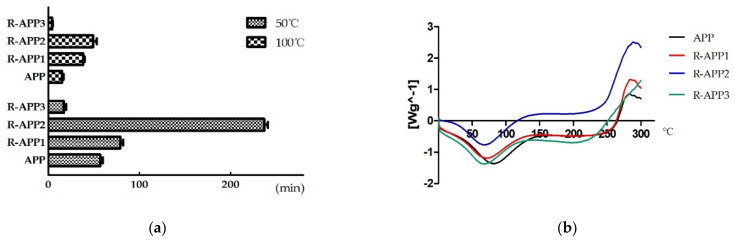
The dissolving time and DSC thermograms of APP and irradiated APP. (**a**) The dissolving time of native APP and irradiated APP at the dose of 10 kGy (R-APP1), 100 kGy (R-APP2), and 1000 kGy (R-APP3); (**b**) the DSC curve of native APP and irradiated APP recorded over a temperature range of 0–300 °C.

The effect of gamma irradiation on the solubility of APPs was determined by the time taken for APPs to dissolve. APPs cannot be completely dissolved at 25 °C. The dissolving time for APPs reduced with the increasing temperature (Figure 2a). It took about 15 min for native APP to dissolve at 100 °C. The solubility of APPs was improved after irradiation at 1000 kGy (R-APP3), which is attributed to the depolymerization of APPs with the breakage of glucosidic bonds and chain scission. The increased solubility was induced by the decrease in inter-chain hydrogen bonds [18]. Incredibly, remarkable extension of the dissolving time of APPs irradiated at 10 kGy (R-APP1) and 100 kGy (R-APP2) was observed. It was inferred that gamma irradiation probably initially caused the branched chain fracture of polysaccharide, followed by full degradation at the high irradiation.

The DSC thermograms were determined to investigate the thermal stability by means of the enthalpy changes of APPs after gamma irradiation (Figure 2b). The APPs were relatively stable below 250 °C, whether with irradiation treatment or not. This is consistent with previous studies [4]. As shown in Figure 1b, two obvious thermal transition peaks were observed in the DSC curves of APPs. A broad endothermic peak at around 70 °C was attributed to the loss of adsorbed and structural water in the polymer [25]. An additional exothermic event was found at around 280 °C in native APPs, which was ascribed to the decomposition and degradation of polymer [21]. The decomposition temperature of R-APP1 and R-APP2 exhibited a remarkable increase in a dose-dependent manner. However, the exothermic peak was not observed in thermograms of R-APP3 below 300 °C, which indicated that R-APP3 required a higher decomposition temperature and had a more stable structure. This suggested that irradiation, especially at high doses, had a significant influence on the thermal stability of APPs. The irradiation of konjac glucomannan without ethanol pretreatment resulted in more stable structures with the higher decomposition temperature [21]. Additionally, the water-soluble polysaccharide from the irradiated mushroom showed an improvement in thermal properties compared with the non-irradiated mushroom samples [26].

**Figure 3:**
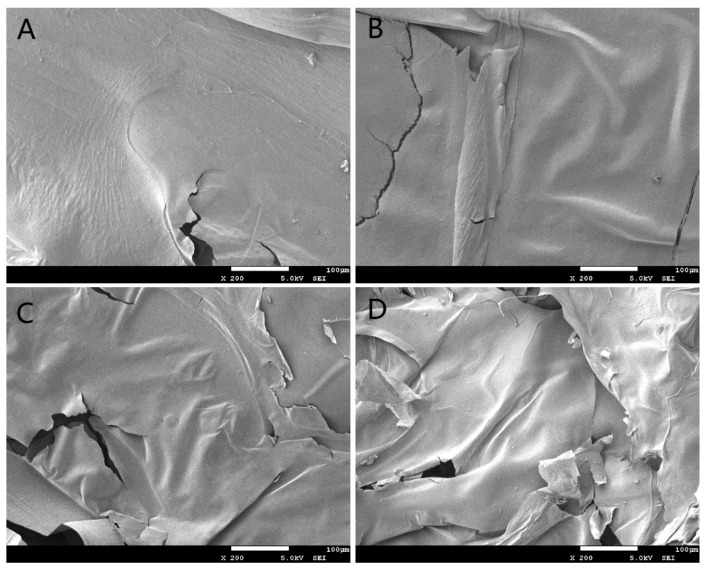
Photomicrograph of native APP and irradiated APP under scanning electron microscopy. (**A**) Native APP; (**B**) 10 kGy; (**C**) 100 kGy; (**D**) 1000 kGy.

Scanning electron microphotographs of APPs and R-APPs are shown in Figure 3; the native APP had a tight structure and a rough surface with flocculent fibers. After irradiation, the R-APPs were crushed into fragments and more fissures were observed. The surface became very smooth. The fibers on the surface disappeared when the APPs were treated with irradiation at applied doses of more than 100 kGy. A smoother and cracked surface has been observed in several polysaccharides after gamma irradiation [18,27]. The clear changes indicated that irradiation had destructive effects on the morphological structure of APPs.

### 2.2. Spectroscopic Analysis of APPs

**Figure 4:**
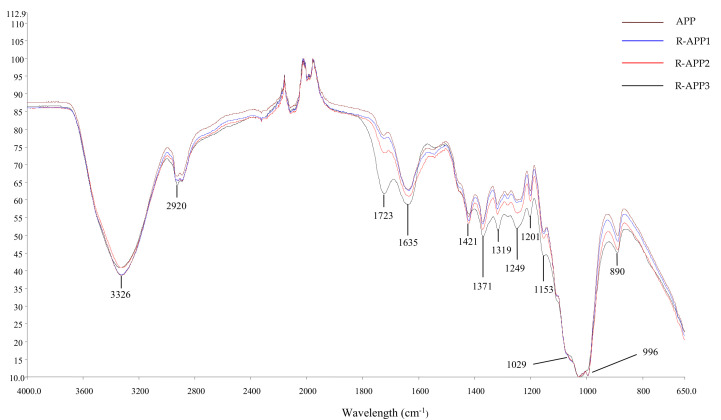
FTIR spectra of native and irradiated APP at doses of 10 kGy (R-APP1), 100 kGy (R-APP2), and 1000 kGy (R-APP3).

Gamma irradiation was used to depolymerize various polysaccharides because of the breaking of glycosidic bonds. FTIR and UV spectroscopy were used to investigate the effect of irradiation on the groups of APPs. As shown in Figure 4, the FTIR spectra of APPs exhibited a strong and broad band at 3326 cm^−1^ due to O-H stretching in hydrogen-bonded hydroxyl groups, which indicated strong inter- and intramolecular interactions in the polysaccharide chain [28]. A sharp peak at 2920 cm^−1^ for C-H stretching and a band at 1421 cm^−1^ confirmed the presence of -CH_2_- or -CH_3_. The peak at around 1635 cm^−1^ was due to the bound water. The absorbance of polysaccharide in the region of 1200–950 cm^−1^ for stretching vibrations of C-O-C and C-O-H were found in the spectra. The characteristic absorptions at 890 cm^−1^ indicated that APPs were the linkage of β-glycosides polysaccharides [13]. After irradiation at different doses, R-APPs had similar spectra characteristics with some remarkable changes in the functional groups, especially for R-APP3. The clear band at 1723 cm^−1^, which was attributed to the absorbance of the carbonyl group [16], strengthened with the increase in irradiation dose in the spectra. Moreover, the intensity band from 1200 to 950 cm^−1^ showed slight differences, varying with the irradiation dose from 0 to 1000 kGy, corresponding to the reduction in C-O-C. This indicated that the irradiation degradation resulted in the newly denoted carbonyl group formation, accompanied by the scission of glycosidic bonds, which was caused by hydroxyl radicals [26]. The formation of a carbonyl group was previously observed in the FTIR and UV spectra of irradiated κ-carrageenan [20].

**Figure 5:**
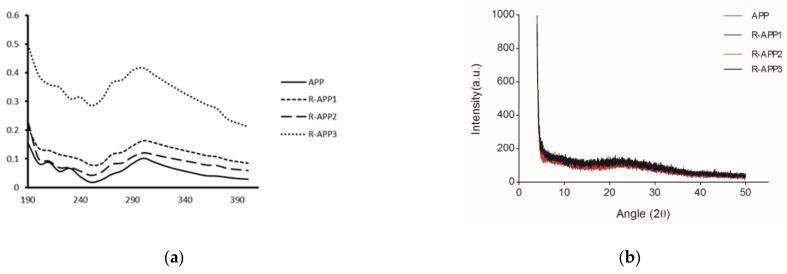
UV–Vis spectra and the X-ray diffraction pattern of native APP and irradiated APP at doses of 10 kGy (R-APP1), 100 kGy (R-APP2), and 1000 kGy (R-APP3). (**a**) UV–Vis spectra; (**b**) X-ray diffraction pattern.

To confirm the carbonyl group formation, the UV–Vis spectra were collected to further elucidate the structural information of APPs and R-APPs (Figure 5a). Absorbance peaks at about 250 nm and 300 nm were clearly observed in the spectra, and were assigned to carbonyl groups and carboxyl group, respectively [16]. After irradiation, the two absorption peaks were found to have strong intensities with the increase in irradiation dose. More reports suggested that gamma irradiation could induce the formation of carbonyl groups of polysaccharides [23]. The X-ray diffraction pattern of APPs is shown in Figure 5b. There were only a few small peaks and no sharp peaks in the diffraction curve of APPs. All APPs exhibited a similar amorphous nature, without any notable changes. This indicated that gamma irradiation had no obvious effect on the crystallization of APPs.

### 2.3. Anti-Hypercholesterolemic Activity of APPs after Gamma Irradiation

**Figure 6:**
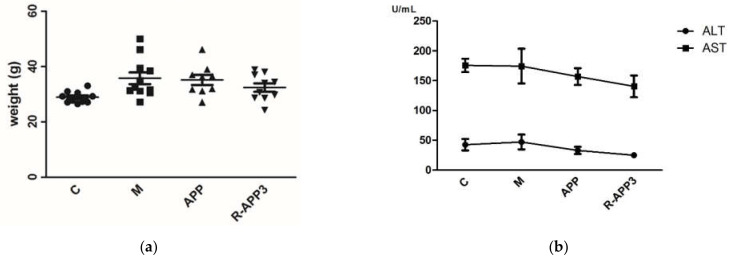
The body weight and serum aminotransferase of normal mice and hypercholesteremic mice. C, normal mice; M, hypercholesteremic mice by intragastric administration of distilled water; APP and R-APP3, hypercholesteremic mice by the intragastric administration of native APP and irradiated APP at a dose of 1000 kGy. (**a**) Body weight of mice, dots with different shapes represented the mice from different groups. (**b**) Serum aminotransferase of mice.

Hypercholesterolemia is one of the major risk factors for recurrent cardiovascular disease, which is associated with the high level of serum cholesterol and lipid abnormalities. Even though lipids are known to be an important energy source, excess intake may induce obesity and hypercholesterolemia [29]. Many polysaccharides have been described to have hypocholesterolemic and hypolipidemic potential in rats and clinical trials, namely, cholesterol bioaccessibility and bioavailability [8,30,31]. Polysaccharide, such as β-glucans, are currently used as hypocholesterolemic food ingredients, which have been accepted by the European Food Safety Agency (EFSA) and the Food and Drug Administration (FDA) [32]. Herein, the hypocholesterolemic effect of APP and its degraded product by irradiation were investigated. After an 8-week induction of a high-fat diet, the body weight gain of hypercholesterolemic mice clearly increased compared with that of the normal mice (Figure 6a). Meanwhile, the levels of serum TG (137%), TC (145%), and LDL-c (167%) were significantly enhanced (*p* < 0.05). Long-term high-fat diet feeding could cause liver damage, with hepatic lipid abnormalities and liver enzyme activity changes [33]. Our results showed that the levels of hepatic TG (175%) and TC (255%) in the hypercholesterolemic mice increased remarkably (Table 2). The intragastric administration of non-irradiated polysaccharides (APPs) restored serum TG to normal levels, decreased the level of serum LDL-c and hepatic TG and TC (Table 2). There was no change in the level of serum of TC after the administration of native APPs. The levels of serum and hepatic TC and TG were restored to normal in hypercholesterolemic mice administrated with APP irradiated at a high dose (R-APP3), as well as the level of serum LDL-c which decreased significantly. Additionally, the slight elevation of serum alanine transferase (ALT) and aspartate transferase (AST) was also observed in hypercholesterolemic mice. R-APP3 treatment could cause serum AST and ALT reductions in hypercholesterolemic mice (Figure 6b). The enhancement of anti-hypercholesterolemic activity of R-APP3 was closely related with changes in its physicochemical and structural properties.

The variable physicochemical properties of polysaccharide, including the molecular weight, water solubility, viscosity, and structure, are responsible for its biological activities. The high-molecular-weight polysaccharide probably had high viscosity and low permeability, which would limit it entering cells to function [12]. Molecular degradation modifications, including physical and chemical degradation, were adopted to reduce the low molecular weight of polysaccharide, thus improving its activity [34,35]. The low-molecular-weight oat beta-glucan produced by irradiation treatment exhibited better antioxidant and anti-proliferative activities than non-irradiated beta-glucan [13]. Enhancement in the anticancer activity of low-molecular-weight fucoidan was obtained by gamma irradiation, which resulted in polysaccharide degradation without changing the functional groups [14]. Generally, high-MW polysaccharide was accompanied by high viscosity and low solubility, which limited its transportation and absorption in the gastrointestinal tract system [36]. Reducing the viscosity and increasing the solubility contributed to improving the biological activity of polysaccharide. The anti-inflammatory activity of polysaccharide from *Schizophyllum* increased by ultrasonic treatment, followed by molecular weight and viscosity decreases [37]. Ultrasonic irradiation could decrease the Mw and intrinsic viscosity of hyaluronic acid (HA) from rooster comb and increase its antioxidant and antiglycation activity [38].

Similarly, the hypocholesterolemic activity of polysaccharide was associated with its physicochemical properties, such as the molecular weight, sugar composition, glycosidic linkages, chain conformation, viscosity, and charge [39]. Many reports showed that the molecular weight and viscosity of polysaccharide played a crucial role in affecting cholesterol homeostasis. β-glucan with a high molecular weight (Mw) and high viscosity could inhibit the diffusion of cholesterol micelles towards the intestinal epithelium membrane, limiting cholesterol bioavailability [40]. However, some results from mice have been controversial. Immerstrand et al. found that the molecular weights and viscous properties of β-glucan products from oat did not influence the hypocholesterolemic effect [41]. Oat β-glucan with a molecular weight of 10 kDa exhibited similar activity to β-glucan, with a molecular weight as high as 2348 kDa. This was most likely a result of different mechanisms of cholesterol-lowering effects. Apart from inhibition of the cholesterol uptake, the main mechanism for polysaccharides is to sequestrate bile acids in the intestine, and thus increase bile acid exclusion into the feces [42]. In addition, the polysaccharide is fermented by gut microbiota to generate short-chain fatty acids (SCFAs), which interfere with cholesterol biosynthesis and convert primary into secondary bile acid, relevant for cholesterol emulsification [43]. Kim et al. noted that low-molecular-weight β-glucan bound more bile acid and produced greater amounts of short-chain fatty acids (SCFAs) than high-Mw β-glucan [44]. In the present study, enhancement of the hypocholesterolemic activity of degraded APP products was associated with the sequestration of bile acid and SCFA production, which was attributed to the lower Mw and viscosity and better solubility. The solubility of polysaccharide has been found to be responsible for its biological activities. Increases in water solubility could improve the biological activity of polysaccharide [45]. Moreover, the enhancement of activity might be related to the structure, such as the formation of carbonyl groups induced by irradiation degradation. The possible hypocholesterolemic mechanism of APP should be further elucidated for better understanding the structure–activity relationships.

## 3. Materials and Methods

### 3.1. Materials and Chemicals

The fresh fruit bodies of *Auricularia polytricha* were collected from the experimental field of the Sichuan Academy of Agricultural Sciences. The fruit bodies were immediately dried in an oven at 50 °C. The reagents for the determination of total cholesterol (TC), triglyceride (TG), and low-density lipoprotein cholesterol (LDL-c) levels were bought from Nanjing Jiancheng Bioengineering Institute (Nanjing, China). The other reagents and chemicals were of analytical grade.

### 3.2. Preparation of Polysaccharide from Auricularia polytricha

The dried fruit bodies were smashed and passed through 160 meshes. Then, 200 g of the power was soaked with 95% ethanol overnight to remove lipids. The residue was boiled in 8000 mL water for three hours. The supernatant was collected and vacuum-concentrated, followed by being dialyzed (Sigma, St. Louis, MO, USA). The polysaccharide complex was precipitated with a 4:1 volume ratio of ethanol and deproteinized by Sevag reagent (N-butanol: chloroform = 4:1). The residue was lyophilized in vacuo to yield 5.2% (*w*/*w*) of crude polysaccharide. The polysaccharide was stored at 4 °C for further use.

The polysaccharide of Auricularia polytricha (APP) was treated by gamma irradiation in a cobalt-60 irradiator (Nordion, Ottawa, Ont, Canada). The levels of irradiation dose were 10 kGy (R-APP1), 100 kGy (R-APP2), and 1000 kGy (R-APP3) at a rate of 50 Gy/min. Dosimetry was performed with a Frickle Dosimeter.

### 3.3. Physicochemical Characteristics of Polysaccharide from Auricularia polytricha

#### 3.3.1. Viscosity

The polysaccharide solution (about 10 mg/mL) after vacuum concentration was gamma-irradiated at the above doses. Then, the viscosities of the native and irradiated polysaccharide solutions were measured with a viscometer (Brookfield, Stoughton, MA, USA.) equipped with an S21 spindle at 160 rpm. The measurements were performed in triplicate at room temperature.

#### 3.3.2. Molecular Weight Determination

The molecular weight of native and irradiated polysaccharides was determined by gel permeation chromatography (Waters 2690, Framingham, MA, USA) equipped with a PL aquagel-OH mixed column and a refractive index detector. Deionized water was used as the mobile phase, with an injection volume of 200 μL (0.5 mg/mL). The various dextran standards were used to plot a calibration curve.

#### 3.3.3. Solubility of APPs

The solubility of APPs with and without irradiation was estimated in water at different temperatures (25, 50, and 100 °C). APPs of 10 mg were weighed and put into 5 mL distilled water in a 10 mL tube. The solution was allowed to infiltrate for 10 min at room temperature, followed by being shaken at 220 rpm in a water bath until they completely dissolved. The time required to dissolve was recorded.

#### 3.3.4. Thermal Analysis

Thermal analysis of the polysaccharides was performed using a Differential Scanning Calorimetry (DSC, METTLER, Zurich, Switzerland). About 5 mg polysaccharides was placed in the aluminum pan and sealed. The experiments were conducted over a temperature range of 0–300 °C at a constant heating rate of 10 °C/min in air.

#### 3.3.5. Scanning Electron Microscopy (SEM)

The morphology of native and irradiated polysaccharides was observed by scanning electron microscopy. The samples were placed on a specimen holder and coated with gold power. The specimens were observed with 400× magnification at an accelerated voltage of 5.0 kV under a field-emission scanning electron microscope (JSM-7500F, JEOL, Tokyo, Japan).

### 3.4. Spectroscopic Characterization

#### 3.4.1. Fourier-Transform Infrared (FTIR) Analysis

In order to investigate the changes in chemical groups of polysaccharides after gamma irradiation, FTIR spectra of APP were analyzed using a Nicolet 6700 Fourier-transform infrared Spectrometer (Thermo, Waltham, MA, USA). Here, 2 mg samples of native and irradiated polysaccharides were mixed with potassium bromide (KBr) and compacted into pellets for spectrometric measurement. The infrared spectra were collected at wavelengths ranging from 4000 cm^−1^ to 650 cm^−1^.

#### 3.4.2. X-ray Diffraction Patterns

X-ray diffraction patterns of the polysaccharides were analyzed by an analytical diffractometer (EMPYREAN, Almelo, Nederland). The operation of the diffractometer was as follows: Cu–Kα radiation at 40 mA, angular range from 3° to 50° (2θ). The scan step was 0.01313 with a counting time of 7.65 s for each step.

#### 3.4.3. UV Spectra

The crude polysaccharides were made into 1 mg/mL aqueous solutions. UV spectra were generated using a UV spectrophotometer (MD 384 Plus, San Francisco, CA, USA) in the range of 190–400 nm.

### 3.5. Anti-Hypercholesterolemic Activities of Polysaccharides from Auricularia polytricha

#### 3.5.1. Animals and Treatment

Forty C57 mice, aged 4 weeks old and weighing 14–16 g, were purchased from Dashuo Biological Technology Co., Ltd. (Chengdu, China). All mice were housed with food and water ad libitum under a 12 h light/dark cycle during the study period.

The hypercholesterolemia model was built as previously described, with slight modifications [8]. After being adaptively fed for one week, all animals were randomly divided into two groups. One group including 10 mice was assigned as the control group (C), which was fed with an ordinary diet (No. D12450B, Huafukang Biological Technology Co., Ltd., Beijing, China). In another group, 30 mice were fed with a high-fat diet (No. D12451, Huafukang Biological Technology Co., Ltd., Beijing, China) which contained 1.5% cholesterol and 34% sucrose for 8 weeks to induce hypercholesterolemia. The tail vein blood of each mouse was collected, and the levels of total cholesterol (TC) were measured to determine the success of the induction hypercholesterolemia model.

Over the next 4 weeks, the hypercholesterolemia mice were randomly divided into three groups (10 in each group): the native polysaccharide group (APP) and the irradiated polysaccharide group (RAPP-3) were fed with a high-fat diet with the intragastric administration of APPs and RAPP-3, respectively, at a dose of 25 mg/kg BW every day; the model group (M) was fed with a high-fat diet and treated with an equal volume of distilled water. Meanwhile, the Control group (C) was fed with a normal diet with the administration of distilled water instead. During the whole experimental period, the body weight of mice was recorded every week.

#### 3.5.2. Measurement of Biochemical Parameters

After 4 weeks of administration, all mice were fasted overnight. The eyeball blood and liver tissue were collected to measure the levels of biochemical parameters. The levels of serum total cholesterol (TC), total triglycerides (TG), low-density lipoprotein cholesterol (LDL-c), and the serum enzyme activities of alanine aminotransferase (ALT) and aspartate aminotransferase (AST) were tested with an Auto-biochemical analyzer (Hitachi 7180, Tokyo, Japan). The levels of hepatic TC and TG were analyzed using commercial kits (Jiancheng Bioengineering Institute, Nanjing, China).

### 3.6. Statistical Analysis

All experiments were performed in triplicate. The data are shown as the mean ± standard derivation (SD) and analyzed with SPSS 19.0 software (SPSS Inc., Chicago, IL, USA). One-way analysis of variance procedures (ANOVA) were applied to determine significant differences between the groups, followed by Dunnett’s *t*-test. *p* < 0.05 was significant.

## 4. Conclusions

Gamma irradiation could cause the degradation of polysaccharide from *A. polytricha*, resulting in a lower molecular weight and viscosity, better solubility, greater thermal stability, and the formation of carbonyl groups at high dose levels. The intragastric administration of APP to hypercholesterolemic rats for 4 weeks decreased serum TG, hepatic TG, and TC. The irradiated APP (R-APP3) exhibited better anti-hypercholesterolemic activity. R-APP3 treatment could significantly decrease the serum TC and LDL-cholesterol, hepatic TG, and TC levels, and restored serum TG to the normal level. The enhancement of anti-hypercholesterolemic activity of irradiated APP (R-APP3) was caused by the changes in physicochemical and structural properties of polysaccharide induced by gamma irradiation. This study might provide some useful insights that polysaccharide from *A. polytricha* could be used as an anti-hypercholesterolemic agent in functional food and pharmaceutical fields. Gamma irradiation is an effective method for high-Mw polymer modification to possess more beneficial biological properties.

## Figures and Tables

**Table 1:** The viscosity and average molecular weight of APPs and irradiated APPs.

Sample	Viscosity (cP)	Mw(kD)	Mn(kD)	Polydispersity (Mw/Mn)
APP	132.65 ± 12.45	6820	1360	5.01
R-APP1	86.54 ± 16.66	2590	306	8.49
R-APP2	15.43 ± 2.75	325	102	3.19
R-APP3	4.76 ± 1.48	34	8.59	3.97

**Table 2:** Serum and hepatic lipid profiles in mice.

Group	Parameters (Units)				
	Serum TC (mmol/L)	Serum TG (mmol/L)	Serum LDL –c (mmol/L)	Hepatic TG (mg/g Pro)	Hepatic TC (mg/g Pro)
C	4.22 ± 0.17 ^b^	1.23 ± 0.04 ^b^	1.22 ± 0.08 ^c^	84.32 ± 7.16 ^c^	33.76 ± 2.23 ^c^
M	5.77 ± 0.18 ^a^	1.78 ± 0.14 ^a^	2.04 ± 0.11 ^a^	147.77 ± 12.32 ^a^	86.32 ± 6.36 ^a^
APP	5.93 ± 0.14 ^a^	1.37 ± 0.09 ^ab^	1.93 ± 0.14 ^ab^	126.86 ± 7.48 ^ab^	76.87 ± 3.68 ^ab^
R-APP3	4.37 ± 0.25 ^b^	1.39 ± 0.07 ^ab^	1.80 ± 0.19 ^b^	101.11 ± 8.02 ^bc^	68.82 ± 3.85 ^b^

Data are expressed as mean ± SD (*n* = 10). Values with different superscript letters within a column are significantly different from each other at *p* < 0.05.

## Data Availability

Data is contained within the article.

## Sample Availability

Samples of the polysaccharides are available from the authors.
